# Myelin replacement triggered by single-cell demyelination in mouse cortex

**DOI:** 10.1038/s41467-020-18632-0

**Published:** 2020-09-29

**Authors:** Nicolas Snaidero, Martina Schifferer, Aleksandra Mezydlo, Bernard Zalc, Martin Kerschensteiner, Thomas Misgeld

**Affiliations:** 1 Institute of Neuronal Cell Biology, Technische Universität München, 80802 Munich, Germany; 2 German Center for Neurodegenerative Diseases (DZNE), 81377 Munich, Germany; 3 Institute of Clinical Neuroimmunology, University Hospital, Ludwig-Maximilians Universität München, 81377 Munich, Germany; 4 Biomedical Center (BMC), Faculty of Medicine, Ludwig-Maximilians Universität München, 82152 Martinsried, Germany; 5 Munich Cluster for Systems Neurology (SyNergy), Munich, Germany; 6 Inserm, CNRS, Institut du Cerveau, Pitié-Salpêtrière Hospital, Sorbonne Université, 75013 Paris, France

**Keywords:** Oligodendrocyte, Myelin biology and repair

## Abstract

Myelin, rather than being a static insulator of axons, is emerging as an active participant in circuit plasticity. This requires precise regulation of oligodendrocyte numbers and myelination patterns. Here, by devising a laser ablation approach of single oligodendrocytes, followed by in vivo imaging and correlated ultrastructural reconstructions, we report that in mouse cortex demyelination as subtle as the loss of a single oligodendrocyte can trigger robust cell replacement and remyelination timed by myelin breakdown. This results in reliable reestablishment of the original myelin pattern along continuously myelinated axons, while in parallel, patchy isolated internodes emerge on previously unmyelinated axons. Therefore, in mammalian cortex, internodes along partially myelinated cortical axons are typically not reestablished, suggesting that the cues that guide patchy myelination are not preserved through cycles of de- and remyelination. In contrast, myelin sheaths forming continuous patterns show remarkable homeostatic resilience and remyelinate with single axon precision.

## Abstract (web summary)

Myelination is a highly regulated phenomenon. Here, the authors show that demyelination induced by the loss of a single oligodendrocyte triggers replenishment of a specific fraction the original myelin and myelination of previously unmyelinated axons in the mouse cortex.

## Introduction

Myelin is a multi-layered membrane sheath around vertebrate axons, which in the central nervous system (CNS) is generated by oligodendrocytes (OLs). It increases conduction velocity and provides metabolic support for axons^1–3^. While myelin was previously perceived as a stable and stereotypical structure^4^, recent work shows that myelination patterns can be dynamic and highly variable, especially outside classical white matter tracts^5,6^. This suggests that myelin could support circuit plasticity and hence contribute to higher brain functions, such as learning and memory. For instance, structural changes in white matter have been associated with task learning in the adult brain^7–9^, and indeed such learning processes require active myelination, as well as oligodendrocyte precursor cell (OPC) proliferation and differentiation^10–14^.

Central to this new paradigm of adaptive myelin plasticity is the unique myelination pattern described in mammalian gray matter including the cortex. In contrast to the myelination patterns in white matter tracts, such gray matter myelin forms a complex sequence with myelinated and unmyelinated stretches resulting in a discontinuous pattern along both excitatory and inhibitory axons, with the latter being more frequently myelinated^6,15–17^. Current data suggest that the editing of such “patchy” myelination patterns involves the emergence of new OLs from non-myelinating precursors. For instance, the number of OLs increases until late adulthood in the cortex^5,18^, but OPC numbers remain stable, implying that they proliferate to match the generation of new myelinating OLs, at least in rodents^19,20^. At the same time, there appears to be a steady, albeit subtle turnover of OLs from the adult cortex. For instance, based on ^14^C incorporation, 2.5% of oligodendrocytes in human adult cortex are renewed per year^21^. In mice, OL turnover might amount to as much as 1.5% per month in the adult motor cortex^22^. While the loss and replacement of individual OLs are unlikely to cause conduction blocks in white matter tracts, the turnover could be detrimental to precise circuit computation, such as spike timing-based plasticity^23,24^.

These findings raise the question of how cortical myelination patterns can be maintained in vivo, given the conflicting demands of participating in ongoing plasticity of neural circuits on the one hand and preserving prior myelin patterns during OL turnover on the other. Indeed, while the patchy appearance of cortical myelin might hint toward a random pattern, the adaptation of myelin sheaths during circuit development and plasticity^25^ suggests that regulatory principles exist to guide both developmental cortical myelination, but also later spontaneous or induced additions of sheaths to cortical myelin. While histological and ultrastructural investigations of mammalian white matter tracts have supported the view that reformed myelin after demyelination can differ substantially from the original ensheathment, e.g., in internode length and thickness^26,27^, recent investigations of the developing zebrafish spinal cord show that primary myelination can resume with precise internode replacement if reset by OL ablation^28^. However, as developmental myelination and remyelination differ substantially^29^, and highly regenerative zebrafish larvae lack a cortex or even a clear-cut white/gray matter divide^30,31^, it remains speculative, whether this tenet of internode restoration applies to myelin replacement in mammalian cortex. Understanding this is particularly important in the context of demyelinating diseases like multiple sclerosis, where extensive cortical demyelination is emerging as a major pathological feature and a central target of remyelination therapies^32–38^.

Here we show that the loss of a single cortical OL triggers a remarkable local response during which newly matured OLs replace the original cell in a homeostatic fashion. These new OLs then restore two-thirds of the original myelin sheaths, with a strong bias toward axons that originally were continuously myelinated. At the same time, axons in the surrounding area receive de novo myelination, mainly as patchy internodes, approximating the original amount of myelin in this cortical area.

## Results

### Targeted oligodendrocyte ablation by focal laser lesion

**Fig. 1:**
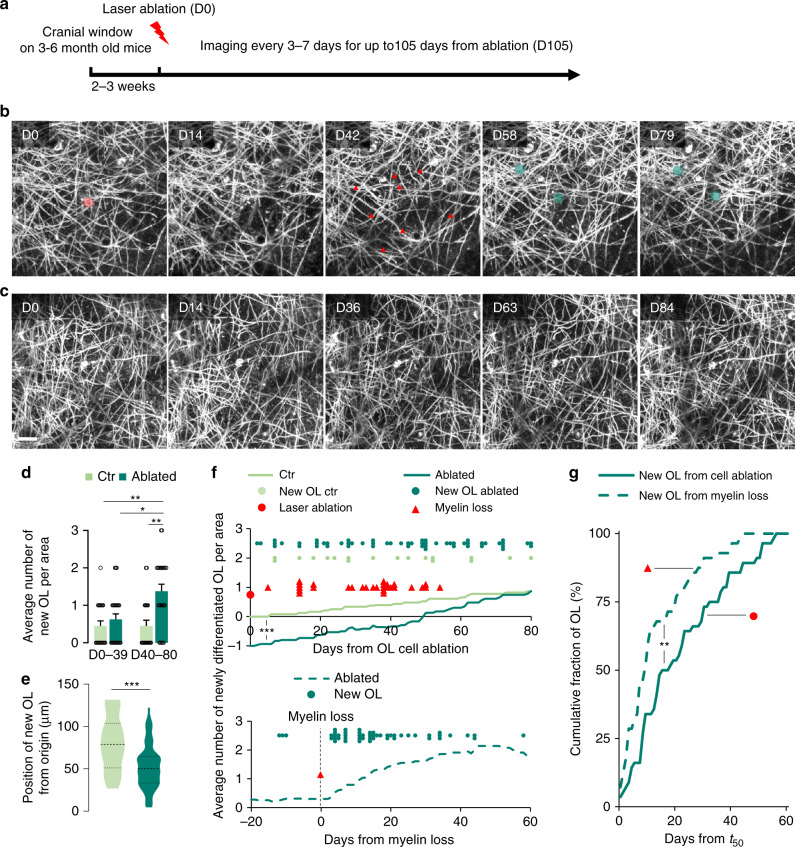
Demyelination due to single OL ablations triggers OPC differentiation in the cortex. **a** Schematic representation of the experiment timeline. **b** Longitudinal in vivo imaging of demyelination after single OL laser ablation (ablated cell highlighted in red at D0 and lost myelin segments marked by red arrowheads at D42) followed by new OL maturation and remyelination in *Plp*:GFP mice (new cells highlighted in green from D58). **c** Longitudinal in vivo imaging of non-ablated control area. Display **b**, **c** Maximum intensity projections of 30 µm. **d** Average number of newly matured OLs per area (*x*/*y*/*z*: 203*203*60 µm^3^) over 80 days for control and ablated areas. Before D40 control: 0.5 ± 0.1; ablated: 0.7 ± 0.1; after D40 control: 0.5 ± 0.2; ablated: 1.4 ± 0.2 (control: *n* = 20 and ablated: *n* = 24 areas from *n* = 10 and *n* = 8 mice per group, respectively; mean ± SEM, Kruskal–Wallis with Dunn’s multiple comparison test). **e** Average distance of the newly matured OLs from the ablated cell or from the center of the control area at D0 (control: 78.9, *n* = 17; ablated: 50.3 µm, *n* = 56; median, two-tailed *t*-test). **f** (upper panel) Timing and distribution of myelin loss and newly matured OLs starting from ablation, imaged in ablated and in control areas, with curves representing the average cumulative number of new OLs per area in ablated and control areas (Kolmogorov–Smirnov test). **f** (lower panel) Timing of newly matured OLs centered on myelin loss in ablated areas, with curve representing the average cumulative number of new OLs per area in ablated areas. **g** Cumulative distribution of new OLs that emerged after *t*_50_ (for definition, see “Methods” section) after either centering on cell ablation (full line) or myelin loss (dashed line; Kolmogorov–Smirnov test). ***P < 0.001, **P < 0.01, *P < 0.05. Scale bar: 20 µm.

To study the effect of losing a single OL on the complex myelin pattern in mouse gray matter, we utilized two-photon laser ablation^28,39^ in 3–6-months old *Plp*:GFP mice^40^ followed by chronic intravital imaging through a cranial window above the somatosensory cortex^41^ (Fig. 1a). In the *Plp*:GFP transgenic line, due to the presence of the PLP peptide signal in the construct upstream of the green fluorescent protein (GFP) coding sequence, the reporter is also inserted in the myelin sheath^42^. The laser ablation technique enabled us to monitor the physiological response to discrete myelin loss in an intact environment up to 105 days (Fig. 1b). To exclude chronic off-target damage of our ablation paradigm, we used AAV-*Mbp*:mem-tdTom injections in *Cx*_*3*_*cr1*^GFP/+^ knock-in mice^43^, allowing simultaneous visualization of OLs and microglia. Laser ablation led to immediate loss of the OL cell body and a swift reaction of resident microglia^44,45^. Still, tissue damage was minimal, resulting in scar-free resolution of the microglial reaction within 24 h (Supplementary Fig. 1a). In addition, after the loss of myelin in the OL ablation setting, axons revealed by sparse neuronal labeling (AAV-*Mbp*:mem-tdTom injections in *Thy1:*GFP^M^ mice^46^) did not show any signs of damage (such as beading or transections) and axonal ultrastructure appeared normal in our correlative electron microscopy (EM) analysis (Supplementary Fig. 2). Therefore, OL laser ablation allowed us to explore single-cell repair strategies of myelin in a minimally altered CNS microenvironment.

### New oligodendrocyte appearance coincides with myelin loss

We performed long-term repetitive imaging following the ablation of mature cortical OLs (Fig. 1 and Supplementary Movie 1). We observed loss of myelin and appearance of one to three newly matured OLs within 80 days after ablation in 95.8% of the studied areas (of these, one cell appeared in 18.2%, two in 50.0%, and three in 31.8% of all areas; Fig. 1b, d). In nearby non-ablated areas (Fig. 1c), the appearance of new OLs was less frequent with 60% of the areas showing newly matured OL (of these, one cell appeared in 50% and two in the other 50% of areas). This baseline rate of OL addition is in line with previous reports^5,47^. Furthermore, on average a single new OL was added by ablation compared to non-ablated control cortex areas (Fig. 1d and Supplementary Fig. 3a) suggesting that the removal of a single mature OL triggered a homeostatic substitution. Indeed, we found many of the new OLs in close proximity to the ablation site within our field of observation and these new OLs appeared significantly closer to the laser-targeting site in the center of each field after ablation compared to “spontaneous” new OLs in controls (Fig. 1e and Supplementary Fig. 3b).

We next monitored the timing of new OL emergence by repetitive intravital imaging. In the absence of laser ablation, new OLs were incorporated at a constant low rate in the adult cortex. However, starting 40 days after ablation, we observed a significant increase of newly matured OLs (Fig. 1d, f). The appearance of these induced new OLs did not seem to be tightly time-locked with the ablation itself, suggesting a different trigger. We noticed that ablation-induced myelin clearance, which was accompanied by a second, but local microglial reaction (Supplementary Fig. 4), was likewise variably delayed between 7 and 55 days post-ablation (Fig. 1f), consistent with previous observations after genetic bulk removal of OLs^48^. We asked, whether centering the emergence of new OLs on the myelin loss time point (Supplementary Fig. 1b), rather than the ablation, would yield a more uniform time course. Indeed, there was a clear increase in the rate of OL insertion within days after myelin loss (Fig. 1f, g). Notably, the slope of the accumulation curve of new OLs was significantly steeper if it was time-locked on the peak of myelin loss (see “Methods” section) rather than on OL ablation (Fig. 1g). This suggests that the appearance of the new OL was triggered by events related to myelin degradation rather than OL death.

### Single oligodendrocyte ablation induces local remyelination

**Fig. 2:**
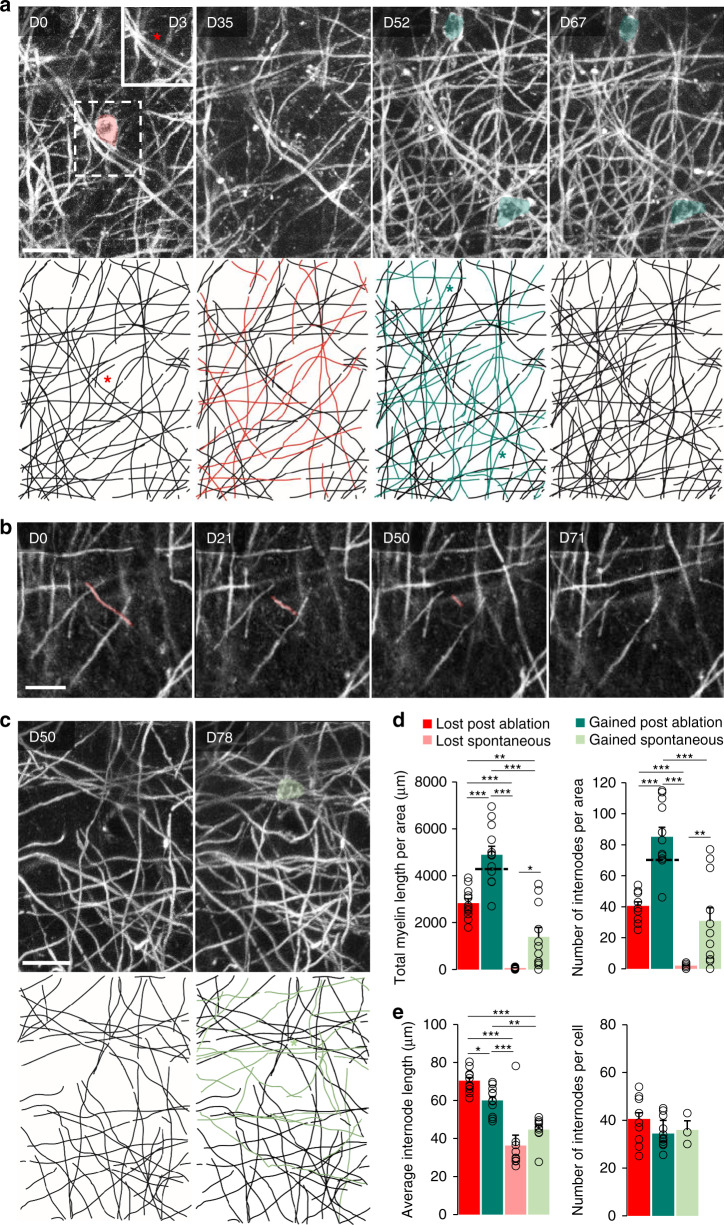
Single OL ablation induces local remyelination. **a** Longitudinal in vivo imaging (upper panel) and manual tracing (lower panel) of an OL cell body and myelin loss (in red) at single internode resolution after OL ablation (at D0, red asterisk), followed by new OL differentiation and remyelination (in dark green and marked by dark green asterisks from D52). Insert: Imaging of the ablated site 3 days post-OL ablation (red star indicates the position of the OL ablated at D0). **b** Spontaneous retraction and loss of a myelin sheath in a non-ablated area. **c** OL cell body and myelin (in light green and marked by a light green asterisk) gained after spontaneous OPC differentiation (D78 after the start of the experiment) in a control area. Display **a**, **c** Maximum intensity projections of 27.5 µm, **b** 4 µm. **d** (left) Total myelin length lost and gained after single OL ablation or in control condition (area *x*/*y*/*z*: 203*203*60 µm^3^; lost post-ablation: 2831 ± 183 µm, gained post-ablation: 4894 ± 361 µm, lost spontaneous: 64 ± 113 µm, gained spontaneous: 1389 ± 401 µm). **d** (right) Average number of lost and gained myelin segments after single OL cell ablation or in the control condition (lost post-ablation: 40.5 ± 2.6, gained post-ablation: 85.1 ± 6.2, lost spontaneous: 1.9 ± 0.4, gained spontaneous: 30.8 ± 8.5). The dashed line on the gained myelin post-ablation displays the level of the lost plus the spontaneously gained fractions. **e** (left) Average internode length of the lost and gained myelin segments after single OL cell ablation or in control condition (lost post-ablation: 70.4 ± 1.6 µm, gained post-ablation: 60 ± 2.0 µm, lost spontaneous: 36.3 ± 5.4 µm, gained spontaneous: 44.7 ± 2.1 µm). **e** (right) Average number of lost and gained myelin segments after single OL cell ablation or in control condition per cell (lost post-ablation: 40.5 ± 2.6, gained post-ablation: 34,4 ± 1.8, gained spontaneous: 36.0 ± 3.8). Statistics for **c**, **d,** and **e** (left) per *x*/*y*/*z*: 203*203*60 µm^3^ area; ablated: *n* = 12 and spontaneous: *n* = 11 areas from 5 and 6 mice per group, respectively; for **e** right: spontaneous: *n* = 3 areas from three animals. Statistics: mean ± SEM, one-way ANOVA with Tukey’s multiple comparisons test. ***P < 0.001, **P < 0.01, *P < 0.05. Scale bars: 20 µm.

Next, we characterized the remyelination events following OL ablation. Our imaging technique allowed us to achieve single internode resolution in vivo for cortical volumes exceeding the typical territory of a single OL (Supplementary Movie 1) and to track internode fate after single OL ablation (Fig. 2a) or during spontaneous sheath loss or OL addition in control areas^5,18,49^ (Fig. 2b, c). First, we investigated the morphology and internodal territory of the new OL in relationship to the original ablated OL. While dense *Plp*:GFP labeling did not allow single OL reconstructions, subtraction of the pre- and post-myelin loss frames enabled reconstructing the ablated OL’s prior territory^39^ and relating it to the new cells’ projections. Compared to the lost territory and taking into account the rate of addition of sheaths formed by spontaneously emerging OLs in control areas, the total myelin length per imaging field increased by 16% after ablation, while the total number of internodes increased by about 20% (Fig. 2d). The number of internodes per OL was not different between the original and newly matured OLs (Fig. 2e). A likely explanation for this apparent excess in internode number and myelin length after ablation is the more central position of the ablation-induced new OLs in the imaged area (which was centered on the ablation site; Fig. 1e and Supplementary Fig. 3b), allowing a more complete assessment of their myelin territories compared to that of spontaneously added new OLs. Together, this suggests a largely homeostatic replacement of sheath numbers after single-cell demyelination. Notably, while on average the internodes that were formed after cortical remyelination were shorter than the pre-existing ones (Fig. 2e), they were longer than the new internodes formed by spontaneously emerging OLs (Fig. 2c–e). This suggests that demyelination frees the axonal surface that is especially supportive of myelination and induces longer internodes than normally formed by spontaneous myelination in the adult cortex.

**Fig. 3:**
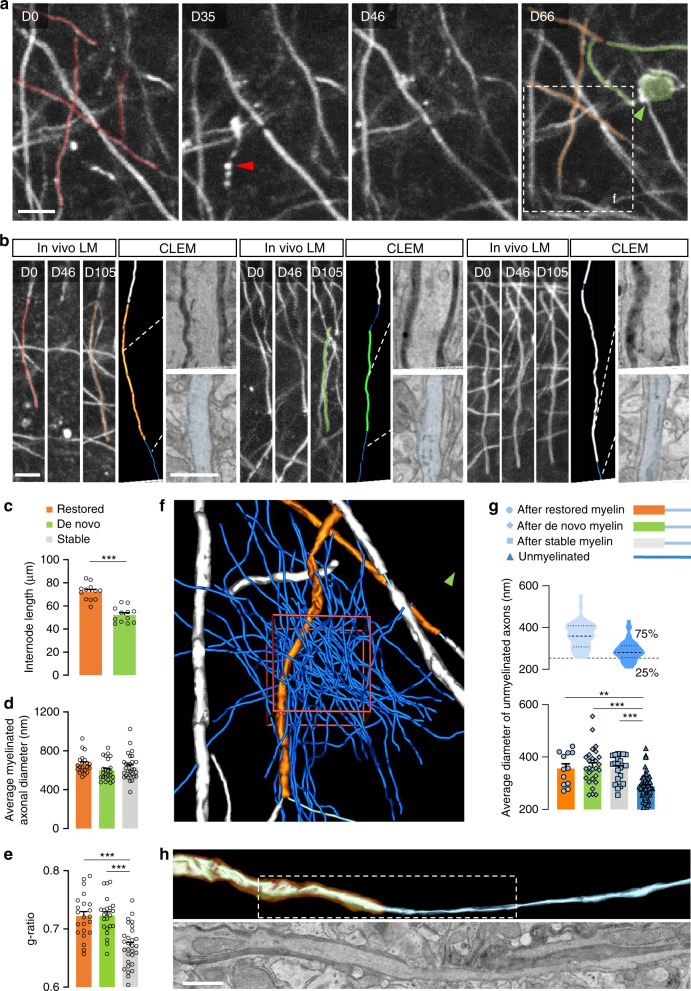
New OLs form internodes that approximate pre-existing myelin. **a** Representative images of longitudinal in vivo imaging allowing the identification of lost (red), degenerating (red arrowhead), restored (orange), and de novo myelinated (green) segments after single OL ablation (newly matured OL: green arrowhead). **b** Representative light microscopy images for each internode fate (restored, de novo, and stable) over the course of a single OL ablation experiment with correlated EM 3D reconstruction and high-resolution scanning electron micrographs of the myelin sheath and underlying axon. Display **a**, **b** Maximum intensity projections of 5 µm. **c** Average length of restored and de novo internodes (restored: 72.1 ± 2.0 µm, de novo: 52.2 ± 1.9 µm; *n* = 12 areas from 5 mice). **d** Average axonal diameter of myelinated segments, measured along, for each fate: restored: 664 ± 21.3 nm, de novo: 598 ± 21.9 nm or stable: 632 ± 24.3 nm (*n* = 21 restored, *n* = 24 de novo, *n* = 29 stable). **e** Average *g*-ratio of restored: 0.72 ± 0.008, de novo: 0.72 ± 0.007 and stable internodes: 0.67 ± 0.006 (*n* = 21 restored, *n* = 24 de novo, *n* = 29 stable). **f** High-resolution EM reconstruction of unmyelinated axons ≥200 nm of diameter neighboring a restored segment within a 10*10*3 µm^3^ volume delimited in red (CLEM of the area displayed in **a**). The green arrow indicates the location of the newly matured OL. **g** Distribution of unmyelinated axon diameters, consecutive to a restored segment: 356 ± 18.9 nm (*n* = 12), de novo: 364 ± 13.7 nm (*n* = 27) or stable: 357 ± 11 nm (*n* = 21) internodes compared to unmyelinated axons within the boxed volume in **f** (288 ± 6.6 nm; *n* = 52). **h** Representative 3D EM reconstruction and corresponding electron micrograph illustrating axonal diameter transition at a restored heminode. Statistics; mean ± SEM; **c** two-tailed *t*-test; **d**, **e** one-way ANOVA with Tukey’s multiple comparisons test; **g** Kruskal–Wallis with Dunn’s multiple comparisons test. ***P < 0.001, **P < 0.01. Scale bars, light micrographs: 10 µm, electron micrographs: 1 µm.

To test this hypothesis directly, we needed to match internodes reliably before and after myelin loss and then relate them to their ultrastructural correlate. Here, we built on a previous study, which showed that in the mouse somatosensory cortex reconstructing less than 40 µm of axonal trajectory suffices (together with tissue landmarks) to discriminate a light microscopically identified axon from all its neighbors in correlated 3D EM volumes^50^. As the average internode length exceeded this “ambiguity” length limit (Fig. 2e), we could reliably follow the fate of single internodes after OL ablation and determine, whether a new internode had restored a lost one or had formed de novo on a previously non-myelinated axon stretch (Fig. 3a). Using this approach, we found that about two-thirds of the original internodes (66.4 ± 2.9%) were restored. Comparing the morphological features of restored and de novo internodes at the cellular and ultrastructural levels (Fig. 3b–e) revealed no apparent differences in myelin compaction and cytoplasmic spaces (Supplementary Fig. 5a, b). Restored internodes were substantially longer than the de novo segments, which were comparable in length to new segments produced by spontaneously emerging OLs (Figs. 2c and 3b, c and Supplementary Fig. 6). For both restored and de novo fractions, the final length of the newly produced myelin sheath was rapidly achieved after the initiation of myelin formation (Supplementary Fig. 7). However, both types of internodes, when we analyzed them at more than 40 days after initiation of myelin formation, had thinner myelin compared to pre-existing internodes, leading to an increased *g*-ratio (Fig. 3e and Supplementary Fig. 5c). Notably, in areas where we could by chance analyze spontaneously formed new myelin sheaths that emerged before ablation-induced myelin loss (which triggers new OL formation), these internodes, even at 90 days after their formation, still appeared to be thinner compared to pre-existing internodes (Supplementary Fig. 5d). Moreover, while previously myelinated axons induced longer internodes, this difference was not driven by axon caliber, as internodes of both classes sheathed axons of similar diameter, which also did not differ from the axons sheathed by unaffected, stable myelin (Fig. 3d and Supplementary Fig. 6). Hence, myelination history rather than the particular geometry of targeted axons determines internode length. Finally, the nodes of the restored sheaths showed a substantial repositioning along the axon, as only 16.0% of nodes appeared in at their original site for internodes that integrate into a continuous pattern and only 29.6% of nodes did so in restored isolated internodes (Supplementary Fig. 8), arguing that many details of an individual OL’s myelin geometry are not fully re-established.

### Remyelination is instructed by pre-ablation myelin pattern

The striking selectivity of the myelination pattern that was induced by OL ablation became apparent once we analyzed the immediate environment of the ablation-induced myelinating internodes in ultrastructurally resolved volumes, revealing the enormous density of potential myelination targets. We reconstructed 52 unmyelinated axons with a caliber ≥200 nm within 300 µm^3^ (*xyz*: 10*10*3 µm^3^) surrounding a remyelinated segment (Fig. 3f). This represented about 10% of all axons in this volume that are “within reach” of the new OL and suitable for myelination based on the previously described 200 nm threshold for CNS myelination^50,51^. We asked whether these “ignored” axons were simply systematically thinner than those chosen for induced myelination. To avoid the confounder that myelination per se could increase axon diameter (Fig. 3h)^52,53^, we compared the axon diameter distribution of unmyelinated axons with the distribution of the “naked” axon parts that adjoined induced myelinated segments, as this likely represents the axon caliber at OL contact. Although the average diameter of the unmyelinated axons was indeed on average slightly smaller, we observed a 75% overlap between these two populations (Fig. 3f, g). We also found no difference in the naked axonal diameter consecutive to a restored, de novo, and stable internode, suggesting that also for these categories, axon diameter is not the driving force (Fig. 3g and Supplementary Fig. 6). From this, we conclude that the new OLs appear to be capable of a highly selective search for specific axons that are preferred targets for cortical remyelination.

**Fig. 4:**
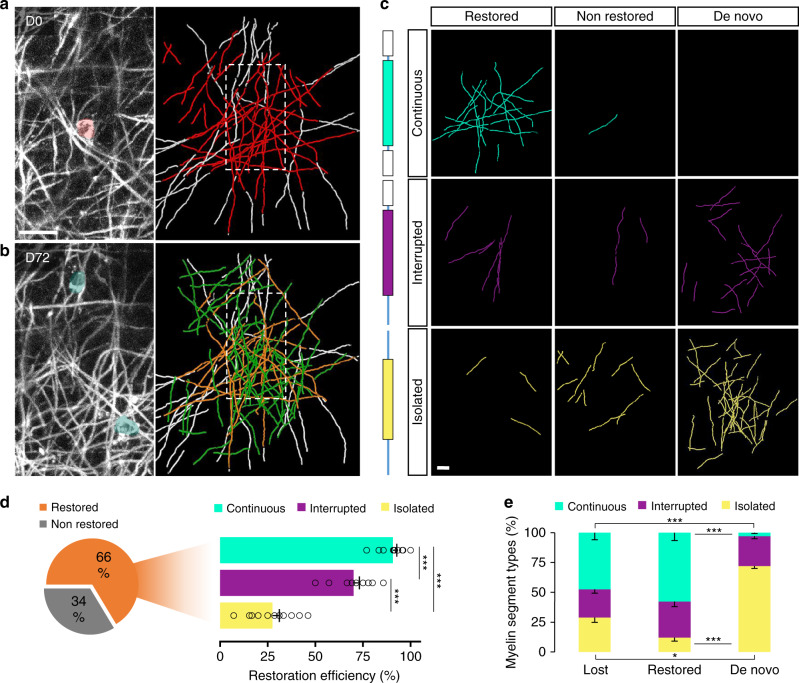
Cortical remyelination efficacy depends on the pre-ablation myelin patterns. **a** (left) Maximum projection of an OL prior to the laser ablation (in red at D0). **a** (right) 3D reconstruction of the lost myelin internodes between D0 and D38 (in red) and their neighboring internodes (in white). The dashed box represents the area displayed in the maximum projection. **b** (left) Maximum projection of a newly matured OL (in green at D72). **b** (right) 3D reconstruction of the gained myelin internodes between D38 and D72 (restored segments in orange and de novo segments in green) and the neighboring internodes of the lost segments between D0 and D38 (in white). The dashed box represents the area displayed in the maximum projection. Display **a**, **b** Maximum intensity projections of 30 µm. **c** Traces of internodes separately displayed according to their post-ablation origin (restored, non-restored and de novo) and their pattern types (continuous, interrupted, and isolated) after single OL ablation. **d** Total and type-specific restoration efficiency from the single OL laser ablation (total: 66.4 ± 2.9%; continuous: 90.7 ± 2.1%, interrupted: 70.1 ± 3.1% and isolated: 27.5 ± 3.6%; *n* = 12 areas from 5 mice, mean ± SEM, one-way ANOVA with Tukey’s multiple comparisons test). **e** Internode type distribution of the lost, restored and de novo myelin fractions (*n* = 12 areas from 5 mice, mean ± SEM, Kruskal–Wallis with Dunn’s multiple comparisons test). ***P < 0.001, *P < 0.05. Scale bar: 20 µm.

To explore this specificity further, we related the probability that an internode was restored to the pre-existing longitudinal myelination patterns along cortical axons. For this purpose, we classified internodes according to criteria that were phenomenologically defined in previous studies^5^. Continuous, where an internode is adjoined by myelin on both sides, interrupted, with neighboring myelin on one side, and isolated (Fig. 4). Based on three-dimensional reconstructions derived from our intravital imaging observations (Fig. 4a, b), we assigned the lost and gained internodes to these longitudinal axonal patterns (Fig. 4c). The degree to which the original myelin territory was restored was strongly influenced by the longitudinal pattern, into which these new internodes integrated: gaps in continuous myelination patterns torn by ablation were usually filled, while isolated segments were rarely restored and interrupted patterns showed an intermediate repair efficiency (Fig. 4d). In accordance with this, when we broke down restored vs. de novo internodes, the former were predominantly part of continuous myelination patterns, while the latter were typically part of isolated patterns (Fig. 4e). The incorporation patterns of the spontaneously added myelin in non-ablated areas was similar to the de novo fraction formed after OL depletion and significantly different from the overall gained myelin in the ablated area (Supplementary Fig. 7a). Taken together these findings indicate that the complex pattern of cortical myelin contains sub-patterns that are preferentially restored and can be read out by emerging OLs with single internode precision. At the same time, these mechanisms do not operate on the majority of cortical axons with patchy myelination, along which the original myelin patterns are erased.

## Discussion

The adult cortex is characterized by a pattern of myelination that at first glance may appear random, but that has been suggested to be specific and functionally important^6,15–17^. We now demonstrate that even loss of a single OL from this pattern can trigger a remarkable local and stereotypical response during which newly matured OLs replace the original cell in a homeostatic fashion. These new OLs then remyelinate two-thirds of the original myelin sheaths, with a strong bias toward axons that originally were myelinated (where >90% of the lost sheath are replaced), as opposed to the isolated internodes that are rarely restored (<30%; Fig. 4). At the same time, axons in the surrounding area receive de novo myelination, mainly as isolated “patchy” internodes, approximating the original amount of myelin in this cortical area. Therefore, the cortical myelin pattern harbors two sub-patterns with differing resilience against small demyelinating perturbations: One, along continuously myelinated axons, that is re-established with single internode precision and another, consisting of isolated internodes, that is not re-established, but replaced by heterotopic patchy myelination mostly of previously unmyelinated axons. This duality allows remyelination, on the one hand, to preserve functionally important structural features on some axons and, on the other hand, to provide new imprints of ongoing plasticity or a substrate for later remodeling on others.

While it has long been known that demyelination is one of the few features of CNS injury that can be spontaneously repaired even in humans, the mechanisms of remyelination have remained controversial^29^, and how precisely such replacement would re-establish the original pattern of myelination is unclear. This question has gained urgency because it is increasingly presumed that myelin supports neuronal network plasticity during learning^7–11,54–56^ using changes in the number, thickness, and spacing of internodes to regulate spike timing^25,57^. If such subtle aspects of myelination patterns indeed contain imprints of past plasticity, major demyelination insults, as they occur in demyelinating conditions such as multiple sclerosis that prominently affect the cortex^58^, might erase such imprints at least transiently. Can such prior patterns in principle be re-established by endogenous or therapeutically supported remyelination, and if so, how precisely? Answering this question is not easy in injury paradigms where most OLs are damaged, as axon loss, glial scaring and local inflammation can obscure remyelination patterns^48,49,59–61^.

We, therefore, established a cortical laser ablation approach that can delete individual OLs with minimal perturbation of the CNS environment. Previously such ablations were used in mice to explore peripheral myelinating cells^39^ and in developing zebrafish to study primary spinal cord myelination^28^. In the zebrafish spinal cord, the precise re-establishment of internodes was observed, irrespective of the surrounding myelination pattern. However, these results do not seem to apply to the mammalian cortex. While our results corroborate that more than 90% of continuously myelinated cortical axons maintains the cues needed to re-establish their myelination, the majority of discontinuously myelinated axons do not (Fig. 4d). Notably, those OLs that remyelinate their original axonal targets do not typically restore a precise positional pattern of the nodes (Supplementary Fig. 8). Indeed, only 8.1% of isolated internodes (29.6% of the 27.5% restored isolated internodes) and 14.5% of internodes that integrate into a continuous pattern (16.0% of the 90.7% of such internodes that are restored), re-establish internode position, as well as presence. Together with the altered g-ratio of internodes after remyelination (Fig. 3), this indicates that in mammalian gray matter, patchy myelination patterns and details of myelin structure that would influence spike timing are mostly erased through a cycle of oligodendrocyte loss and replacement.

The following principles appear to govern the replacement of lost OLs and myelin: First, ablation inducesa homeostatic increase in the generation of new OLs. The timing of this OL differentiation is not related to the death of the ablated OL per se (which was cleared within 24 h of ablation), but rather to the following demyelination, which was delayed by as many as 50 days (average ∼32 days). This is in line with other OL ablation paradigms, where myelin, which also on the protein level has an extremely long half-life^62^, can survive long after OLs have died^48^. In our experiments, we observed that the OL processes resealed rapidly after cell body ablation (Supplementary Fig. 1), then maintained their morphology for weeks before removal by resident microglia (Supplementary Fig. 4), which was consistently followed by OL replacement typically within 2 weeks (*t*_50_ = 9 days). Therefore, it appears that myelin removal triggers OL maturation and remyelination, supporting the notion that myelin removal is a controlled process that can initiate subsequent repair^63,64^.

Second, there appears to be a homeostatic re-establishment of local myelin amount if one considers the spontaneous background addition of OL that occurs at the investigated ages of mice (ref. ^5^ and this study, Fig. 1f). Indeed, each individual new OL finally established a morphology (e.g. number of internodes) that on average matches that of the pre-existing OL population (Fig. 2e, right). As ample additional axons of suitable diameter can be found within the range of an OL’s processes (Fig. 3f, g), this suggests that there are OL-intrinsic components that restrict myelination capacity. The detailed morphology of the newly formed internodes depended on the type of myelination pattern that the new internodes integrated into (Fig. 2e, left and Supplementary Fig. 6a, b): If the new internode re-established a continuous pattern that had been interrupted by OL ablation, the gap was typically filled and the resulting new internode was comparatively long. If, however, a new “patch” of isolated myelin was formed, the internodes were shorter—essentially matching the length characteristics that pre-existed for these classes of internodes. Notably, however, the thickness of myelin and the position of nodes were not preserved, with that myelin being thinner and nodes typically displaced. According to a limited analysis that we could perform in our correlated EM data set, also spontaneously added internodes appeared to be thinner and shorter than ones that existed at the beginning of the experiment (Supplementary Fig. 5d)—suggesting that this may simply be a feature of myelin sheaths that have formed recently or in relatively mature cortex. For instance, signals on the axonal surface that support myelination^65,66^ might be downregulated as the cortex matures or the density of topographical “barriers” that prevent internode elongation, such as synapses and branch points^6,15,16^, might increase. In addition, we did not find any evidence for the elaboration of new internodes by pre-existing OLs in response to ablation (e.g. internodes that could be traced to a pre-existing OL or that appeared in spatial or temporal isolation; Supplementary Fig. 7), suggesting that the previous descriptions of such a remyelination mechanism^61,67,68^ might be restricted to the diseased brain or sublethal injury of oligodendrocytes^69^.

Third, ablation resulted in patches of myelin on axons that were originally not myelinated, i.e., that are “heterotopic” with regards to the original pattern of myelination. The explanation for these heterotopic internodes could be twofold. On the one hand, they could simply represent a random excess due to the OL’s “drive” to myelinate, as also evidenced by the propensity of OLs to myelinate inert substrates in the absence of alternatives^70^. This would imply that the myelinated axons are not in need or even especially suitable for myelination, but are “off-target”. On the other hand, the de novo internodes could be the imprint of the OL’s search for the “right” axons that require myelination. In any case, such newly myelinated axons could serve as the substrate for later plasticity-related modifications. These could result in addition, but also in retraction of internodes, which appear to be relatively common in specific settings^61,71^. On the time-scale of our follow-up (on average 81.1 ± 1.1 days), however pruning of fully differentiated internodes was rather rare (about 1.9 ± 0.4 segments per area), in line with other reports from mice^5,18,49^. This indicates that it is uncommon for cortical myelin to undergo remodeling at the level of single internodes. Rather it could be the exchange of individual OLs that occurs physiologically in both rodents and humans^21,22^, which allows adaptation of cortical myelination patterns.

Fourth, our data show that maturing OLs in the adult cortex have the ability to efficiently find myelination targets in a vast “ocean” of axons. Indeed, recent connectomics reconstructions of cortex showed that 220 axons traverse within a volume of 125 µm^3^ ^50^ when the volume covered by a typical layer 1 OL alone is substantially larger (∼5000-fold) than this^22^. Our own ultrastructural volume reconstructions corroborate this view, showing that a remyelinating OL ignores an enormous number of potential axonal targets, even if their diameters are intrinsically suitable for myelination. This is remarkable in light of the above-cited fact that OLs readily myelinate inert fibers of suitable diameter in vitro^70,72^. It also implies that there likely are three populations of cortical axons that new OLs manage to identify: a few axons that require obligatory myelination (and as a result re-establish their continuous myelination pattern), many axons that actively suppress myelination and remain unmyelinated^66^, and the remaining group of discontinuously myelinated axons that do not strongly enforce either outcome and as a result commonly change their myelination pattern after a cycle of de- and remyelination. The later finding implies that the dominant “patchy” pattern of cortical myelination in the mammalian cortex would be perturbed even after minimal OL loss, suggesting that little long-term information storage can happen in these patterns and that such information would be comprehensively lost after pathological demyelination, even if remyelination ensues.

Overall, our experiments reveal that while OL numbers, myelin content, and continuous myelination patterns are restored, the detailed geometry of internodes is not. This implies that “classic” functions of myelin, such as saltatory conduction and metabolic support are likely restored, but the timing of action potentials might not be preserved. At the same time, the apparently random pattern of cortical myelin contains both highly regulated, but also spurious sub-patterns, which differ profoundly in their resilience even against minimal demyelination.

In summary, our results reveal the degree of fidelity with which remyelination can naturally happen in the mammalian cortex. This is critical information for developing efficient, but at the same time sufficiently precise therapeutic interventions to reverse pathological cortical demyelination^33^.

## Methods

### Transgenic animals and virus labeling

Experiments were performed on transgenic mice that express GFP fused to exon 1, intron 1, and the first 37 bp of exon 2 of the PLP gene driven by the PLP promotor on a C57BL/6 background (in this study called *Plp*:GFP mice)^42^ and *Cx*_*3*_*cr1*^GFP/+^ knock-in mice^43^, as well as *Thy1*:GFP^M^ transgenic mice^46^ bred in our animal facilities. Both female and male animals from 3 to 6 months of age were included in experiments. The animals were housed at a temperature of 22 ± 2 °C with 50 ± 15% of humidity level and under a light/dark cycles of 12 h. For viral labeling of OLs, a recombinant, replication-deficient AAV (AAV-*Mbp*:mem-tdTom; 10^12^ titer, 0.5 µl in Ringer’s solution) was stereotactically injected (coordinates: 2/−2 mm from bregma, 0.2 mm depth) using an ultrathin pulled glass pipette via a 4 mm (diameter) craniotomy performed 14–21 days before the first imaging session.

All animal experiments were performed in accordance with the regulations of the relevant animal welfare acts (TierSchG) and protocols approved by the respective regional authorities (Regierung von Oberbayern).

### Cranial window surgery

To gain optical access to the cortex for monitoring myelin morphology over time, we performed a craniotomy and implanted a cranial window above the somatosensory cortex of the animals as previously described^41^. In brief, mice were anaesthetized with a mixture of medetomidine (0.5 mg/kg), midazolam (5 mg/kg) and fentanyl (0.05 mg/kg) intraperitoneally. The craniotomy was performed using a 0.5 mm stainless steel drill head (Meisinger) and a cranial window of 4 mm of diameter was positioned and fixed with dental cement. Mice were given buprenorphine (0.05–0.1 mg/kg) for analgesia every 8 h on the days following the surgery.

### In vivo imaging

Chronic in vivo imaging of the layer 1 OLs and myelin segments was initiated 14-21 days after the implantation of the cranial window on *Plp*:GFP animals and was performed using an Olympus FV-RS microscope (Olympus, Japan) equipped with a femtosecond pulsed Ti:Sapphire laser (Mai Tai HP-DS, Spectra-Physics) at a maximum power of 30 mW (measured the back focal plane). Imaging was performed with a resonant scanner using 16× averaging. On the imaging days, animals were anesthetized starting with 2% isoflurane, placed on the imaging stage, and provided with a constant flow of 1.2–1.5% isoflurane for the rest of the imaging period. The physiological state of the animals was continuously monitored with a MouseOx system (Starr Life Science Corp) equipped with a thigh sensor to assess the depth of the anesthesia, oxygenation of the blood, and heart rate. On the first imaging day, several areas (5–8; *x*/*y*/*z*: 203/203/60 µm^3^) randomly chosen throughout the somatosensory cortex were mapped and imaged with a pixel size of 0.4 µm in *x*/*y* and 0.5 µm in *z*. One OL per non-control area was then ablated by targeting a couple of pixels at the center of the cell (920 nm, 80–120 mW for 1–2 s in tornado mode). The area was controlled 3 days post-ablation to confirm OL death. The same areas were then imaged every 3–7 days for up to 105 days. Control areas were imaged without ablation for the same period. We observed no signs of photo-damage in both ablated and control areas over the imaging period using these imaging conditions. Areas showing deterioration of the imaging quality throughout the chronic imaging were excluded from the analysis.

In experiments where transgenic mice labeling axons and microglia were used, the OLs were labeled by AAV-*Mbp*:mem-tdTom.

### AAV vector generation and virus production

An AAV-mem-tdTomato plasmid in which reporter gene expression was controlled by the *Mbp* promoter (AAV-*Mbp:*mem-tdTomato) was generated by replacing the *Cmv* promoter in the AAV backbone plasmid (AAV-*Cmv*:mem-tdTomato) with the first 1.3 kb of the *Mbp* promoter. A sequence of 35 amino acids of zebrafish Gap43, fused at the N terminal to TdTomato, guides the protein to the plasma membrane as reported before^73^. In short, AAV-*Cmv*:mem-tdTomato containing bovine growth hormone polyadenylation sequence (bGHpolyA) flanked by AAV2 inverted terminal repeats, was digested with *Age*I-HF/*Bmt*I-HF to replace the *Cmv* promoter with 1.3 kb *Mbp* gene excised with *Sal*I-HF/*Mlu*I-HF from AAV-*Mbp*. Both, the backbone and the insert were blunted with DNA polymerase I (Klenow)/dNTPs and further ligated by T4 ligase in T4 ilgase buffer. A construct with a correctly oriented insert was introduced to competent cells through electroporation. Plasmid purification was performed with a Qiagen Plasmid Maxi Kit according to the attached protocol. AAV vector packaging was performed using human embryonic kidney 293 (HEK 293) cells as described before^74^. Briefly, HEK 293 cells were transfected with pAD-helper, AAV-capsid, and AAV-construct (molar ratio 3:2:5) using the RPMI:PEI incubation protocol. AAV vector was harvested from the supernatant (with PEG solution) and the pellet. Freeze/thaw cycles were utilized to lyse the cells. Any residual DNA from the packaging process was degraded with benzonase before proceeding to the purification step. Virus was purified using iodixanol gradient ultracentrifugation and concentrated by subsequent incubation/centrifugation with formulation buffer (Pluronic-F68 0.001%). We obtained ~300 μl of the virus, which was stored in small aliquots at −80 °C. Genomic titers were ~2.5 × 10^13^.

### Correlative light-to-electron microscopy

After the final imaging session, animals were killed by isoflurane overdose and directly perfused with 4% paraformaldehyde (EMS) and 2.5% glutaraldehyde (EMS) in phosphate-buffered saline (EMS). After fixation, the brains where re-positioned under the microscope using the same orientation as set during the in vivo imaging. Overview images allowed precise identification of the previously chronically imaged areas. For correlated light and electron microscopy (CLEM), large asymmetric near-infrared branding (NIRB) marks^75^ were performed 100–200 µm away from the outermost edges of the imaged areas leading to the marking of a large field (*x*/*y*: ∼1.5*1.5 mm^2^) containing the imaged areas. The tissue was then dissected following these large asymmetric NIRB traces allowing the unambiguous orientation of the areas within the tissue block and further processed for EM imaging.

The en bloc staining protocol was adapted from Hua et al.^76^. Briefly, the tissue was initially post-fixed using 2% osmium tetroxide (EMS) in 0.1 M sodium cacodylate (Science Services) buffer (pH 7.4) that was replaced by 2% potassium ferricyanide (Sigma) in the same buffer. After three washing steps in buffer and water, the staining was enhanced by reaction with 1% thiocarbohydrazide (Sigma) for 45 min at 50 °C. The tissue was washed in water and incubated in 2% aqueous osmium tetroxide (EMS). All osmium incubation steps were carried out over 90 min with substitution by fresh reagents after 45 min, respectively. To intensify the staining further, 2% aqueous uranyl acetate (EMS) was applied overnight at 4 °C and subsequently warmed to 50 °C for 2 h. The samples were dehydrated in an ascending ethanol series and infiltrated with LX112 (LADD). In order to facilitate trimming and sectioning at an angle parallel to the live imaging plane, the sample was oriented with the pial side parallel to the block tip and cured for 48 h.

The block was trimmed by 200 µm at a 90° angle on each side using a TRIM90 diamond knife (Diatome) on an ATUMtome (Powertome, RMC). Consecutive sections were taken with a 35° ultra diamond knife (Diatome) at a nominal cutting thickness of 75 nm and collected on freshly plasma-treated (custom-built, based on Pelco easiGlow, adopted from Mark Terasaki, U. Connecticut), carbon-coated (provided by Richard Schalek and Jeff Lichtman, Harvard U.) Kapton tape^77^. The area of interest was pre-estimated during sectioning according to endogenous (e.g., blood vessel geometry) and NIRB marks. The corresponding tape region with sections was assembled on the adhesive carbon tape (Science Services) mounted onto 4-inch silicon wafers (Siegert Wafer). Kapton and silicon were connected by adhesive carbon tape strips (Science Services) for grounding.

EM micrographs were acquired on a Crossbeam Gemini 340 SEM (Zeiss) with a four-quadrant backscatter detector at 8 kV. In ATLAS5 Array Tomography (Fibics), the whole wafer area was scanned at 2000–6000 nm/pixel to generate an overview map. In total, 391 sections were selected and imaged at 200*200 nm^2^ and a smaller ROI selected for another imaging round at 50*50 nm^2^. The region of interest was relocated based on this image set compared to the live imaging data. The final volume (370*350*47 µm^3^) was acquired with 6.4 µs dwell time at a voxel size of 12*12*150 nm^3^ (every second section). The high-resolution volumes were generated from several image sets of 35–70*35-85*3 µm^3^, at 3*3*∼75 nm^3^ voxel size. In the samples, where correlative electron microscopy was used, the identification of the nodal regions by light microscopy was verified by ultrastructure, as we also did previously^78^.

### Image processing and analysis

Post-processing of the presented two-photon imaging was done using the open-source image analysis software, ImageJ/Fiji (version v1.53c—http://fiji.sc)^79^; brightness and contrast were adjusted, maximum projections were performed for display purpose (see figure legends for details), and the time-points represented in the figures were aligned using the StackReg plugin^80^.

The analysis of the cumulative number of newly differentiated OLs from ablation and in control was performed on in vivo recordings ranging from 29 to 80 days (on average: 72.3 ± 2.9 days for ablated and 74 ± 2.6 days for the control—SEM). In order to statistically compare the variance in time with which of new OLs appeared in ablated areas either in relation to the time point of ablation or the time point of myelin loss, we for both curves (as they lacked a sharp plateau) calculated the time *t*_50_ at which 50% of new OLs had formed after the relevant index event (ablation or myelin loss, respectively). For each new OL, the time span (as a positive number) to *t*_50_ was calculated, either in the ablation or the myelin loss reference time frame. The resulting time spans were then plotted as a cumulative percentage curve to show that when the analysis was centered on myelin loss the time variance of the emergence of new OLs was significantly smaller (Kolmogorov–Smirnov test) than when centered on the ablation time point. The analysis of the lost and gained number and length of myelin segments in the control areas was performed at 81.1 ± 1.1 days (SEM). The analysis of the lost and gained number and length of myelin segments after ablation was performed at 40.5 ± 5.5 days after myelin loss and 21.6 ± 3.3 days after new OLs appeared. Correlative electron microscopy was carried out on samples 105 days post laser ablation.

The number of myelin sheath per cell was measured on ablated OLs and on newly appearing OLs positioned more than 50 µm away from the imaging area edges.

To analyze the position of newly matured OLs in ablated conditions the reference point was set to the center of the ablated cell and for the control areas at the center of the imaged area at D0.

To analyze the length and type of myelin sheaths over the ablation experiments, 3D reconstructions of lost and gained myelin segment were performed within the acquired chronic in vivo imaging volumes using the simple neurite tracing plugin of Fiji. Internodes ending outside of the imaging area were used in the remyelination efficiency, the total number, and the total length of gained/lost segments quantifications, but excluded from the segment type quantification and length per segment type measurements. The length of lost segments was determined based on the initial imaging at D0 and the length of gained internode was determined at the end of the experiment (unless otherwise indicated).

The myelin loss time point was defined at the peak of the observed sheath loss (sampled at a rate of one image every 7 ± 1 days). The volume covered by an OL in layer 1 of the somatosensory cortex was approximated using the volume of a cylinder of 150 µm of diameter (span of the OL in *x*/*y*) and 40 µm in height (span of the OL in *z*) in line with previously published data^22^.

For the 3D EM reconstructions, the images were aligned by a sequence of automatic and manual processing steps in Fiji TrakEM2^81^. IMOD software (version 4.7) was then used for the manual segmentation, tracing, and reconstructions of the CLEM volumes^82^. *G*-ratio measurements were performed in three different places along the central section of the internodes. The myelinated axonal diameter measurements were performed in three to five places (depending on the length of the internode) along the entire length of the internode. The measurements of the unmyelinated axonal diameter section were performed in three places within 30 µm of the neighboring myelin heminode. The diameter measurements of the unmyelinated axons from the high-resolution EM volumes were performed in three places along the reconstructed axon. In Supplementary Fig. 8, the direction of node translation was defined to be positive if the motion occurred toward the restored or non-remyelinated segment.

### Statistical analysis

Sample sizes were chosen according to previous in vivo imaging studies^5,18,49,61^. Statistical significance was calculated with Prism (versions 8.0) using *t*-test, multiple *t*-tests with Bonferroni–Dunn correction or ANOVA and Tukey’s multiple comparison test (where normal distribution could be assumed by D’Agostino & Pearson or Shapiro–Wilk tests) or Mann–Whitney *U*-test or Kruskal–Wallis and Dunn’s multiple comparisons test (where non-normal distribution was suspected and confirmed by Shapiro–Wilk test) as described in the figure legends. The cumulative distributions were tested with the Kolmogorov–Smirnov test.

### Reporting summary

Further information on research design is available in the Nature Research Reporting Summary linked to this article.

## Supplementary information

Supplementary information

Description of Additional Supplementary Files

Intravital imaging of cortical OL in adult mouse.

Reporting Summary

## Data Availability

The *Plp*:GFP mouse line was generated by B.Z. to whom requests for the mouse line have to be directed. *Cx3cr1*^GFP/+^ and *Thy1*-GFP^M^ mice are available from Jackson Laboratory (JAX 005582 and 007788, respectively). The plasmids to generate AAV-*Mbp*:mem-tdTom can be requested from the authors. The full data sets generated during and/or analyzed during the current study are available from the corresponding author on reasonable request. Source data are provided with this paper.

## Source data

Source Data
