# Supplementary material for: Myelin replacement triggered by single-cell demyelination in mouse cortex
Source: Nat Commun. 2020 Sep 29;11:4901. doi: 10.1038/s41467-020-18632-0 (PMC7525521; doi:10.1038/s41467-020-18632-0)
Supplement: Supplementary file 1 — Supplementary information [file 41467_2020_18632_MOESM1_ESM.pdf]

SUPPLEMENTARY INFORMATION

Myelin replacement triggered by single-cell demyelination in mouse cortex

Snaidero et al.

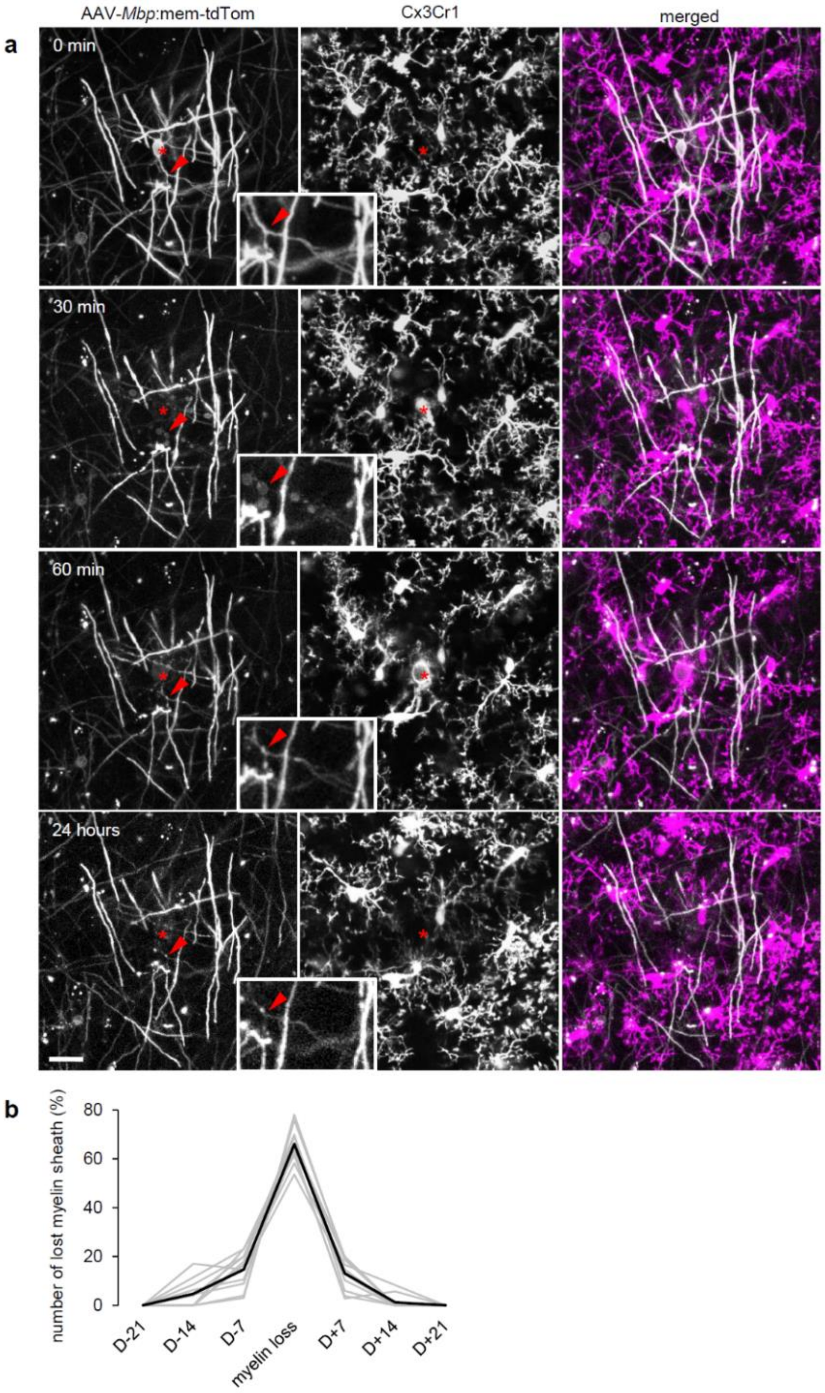

Supplementary Figure 1 | Microglial response to a single OL laser ablation.

a: Intravital imaging of a cortical OL laser ablation with labelled resident microglial cells (*$Cx_3cr1^{GFP/+}$* mouse). Cortical OLs are labelled by injection of AAV-*Mbp*:mem-tdTom. 0 minutes illustrate the pre ablation state. Thirty minutes post ablation, neighboring microglial cells start polarizing toward the ablated OL and by 60 minutes the cell body is surrounded by the neighboring microglial end feet (red star designate the ablated OL soma position). 24 hours post ablation no microglial scar was observed, and the ablated cells internodes persisted with preserved connecting processes (red arrowheads, **inserts**). **b**: Distribution of myelin sheath loss from the defined myelin loss time point (grey curves display myelin loss in single ablated areas, black curve displays the average). Display: 10 µm maximum intensity projection. Scale bar: 20µm.

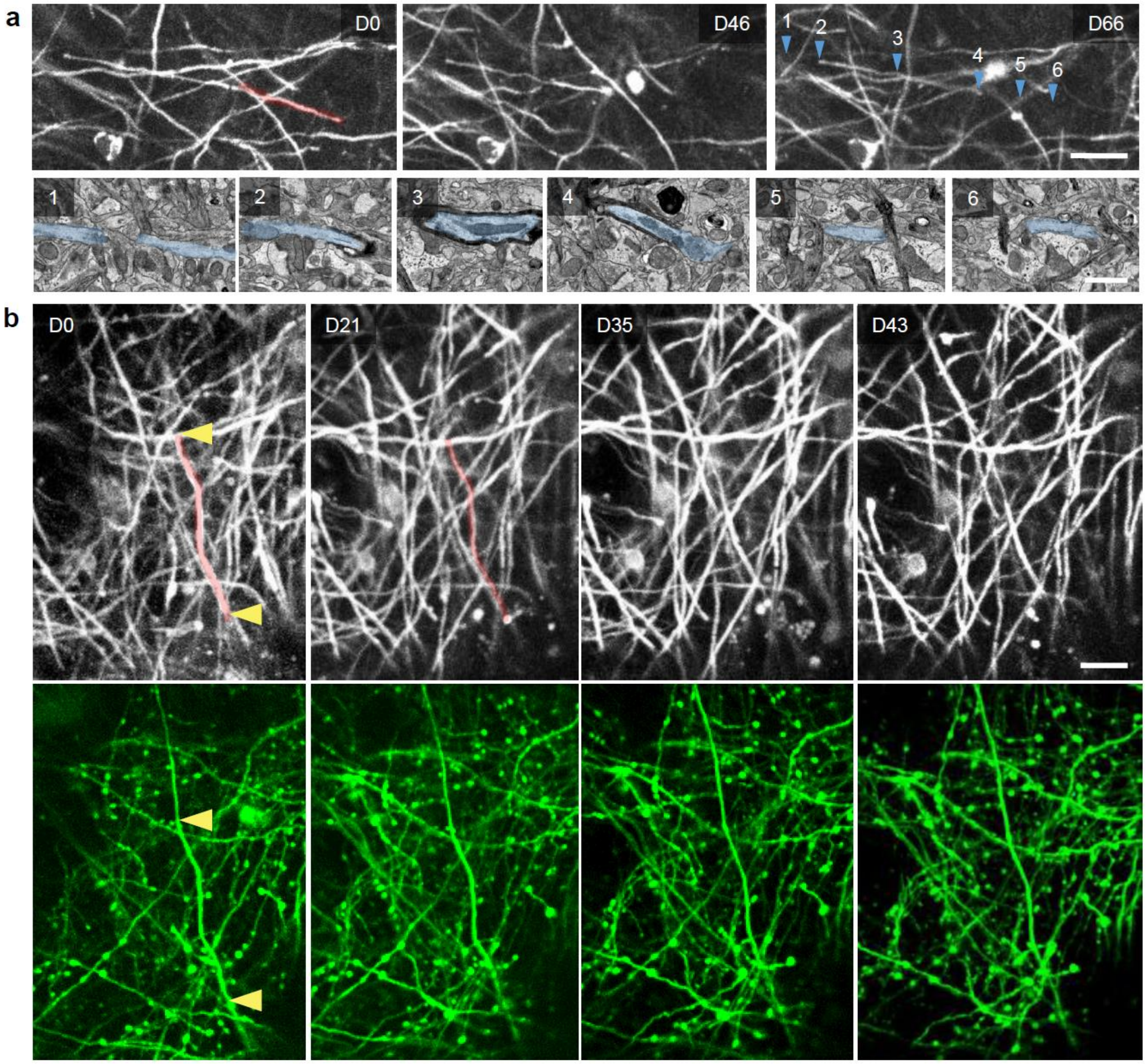

Supplementary Figure 2 | Unaltered axonal morphology after single OL ablation and demyelination.

a, upper panel: Longitudinal in vivo imaging of a demyelination event without remyelination (highlighted in red). **a, lower panel:** Correlative electron micrographs of the partially myelinated axon (blue) displayed in the upper panel. The unmyelinated (1), nodal (2, 4), internodal (3) and non remyelinated (5, 6) portions of the axon are displayed from the areas pointed out at D66 (blue arrowheads). Display: 2 µm maximum intensity projection. Displayed example from one correlative light to electron microscopy experiment. **b**: Longitudinal in vivo imaging of axons near the OL ablation site until demyelination (**upper panel**: myelin labeling by injection of AAV-*Mbp*:mem-tdTom; **lower panel**: sparse axonal labeling in the *Thy1*:GFP^{M} line). The lost myelin segment is highlighted in red and the nodal regions by yellow arrowheads. Representative example from 7 segments (n=3). Display: 2 µm maximum intensity projection. Scale bar, light micrographs: 20 µm, electron micrographs: 1 µm.

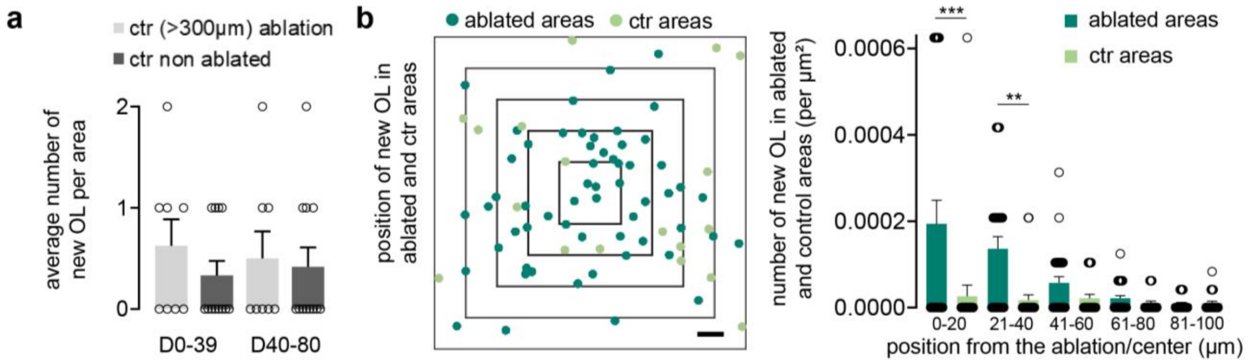

Supplementary Figure 3 | Spatiotemporal and morphological features of newly matured OLs.

a: Average number of newly matured OL per area over 80 days for control areas in animals where laser ablation was performed—ctr ablated— (areas centered >300 µm away from the ablated cells) and in control animals where no OL ablation was performed—ctr non ablated—(ctr ablated: n=8 and ctr non ablated: n = 12 areas from 5 and 2 mice per group, respectively; mean ± SEM, Kruskal-Wallis with Dunn's multiple comparisons test). **b, left**: Position of newly matured OLs in ablated and control areas centered on the ablated cell or the center of the field of view, respectively, at D0. **b, right**: Distribution of newly matured OLs per µm² per area until final time point (control: n=20 and ablated: n=24 areas from n=10 and n=8 mice per group, respectively; mean ± SEM, multiple t-tests with Bonferroni.Dunn's correction). Scale bar: 20 µm.

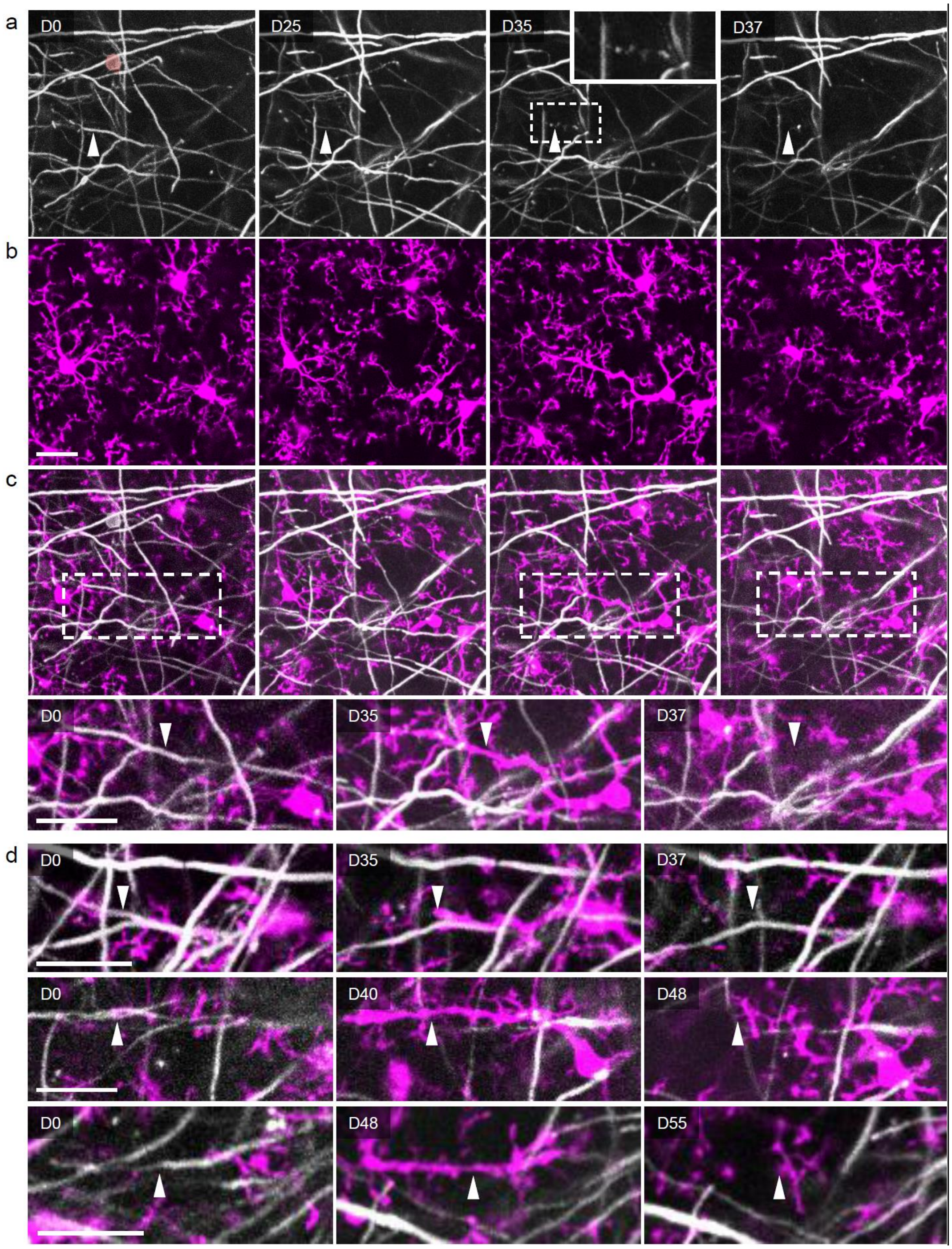

Supplementary Figure 4 | Removal of myelin fragments by neighboring microglia.

a: Longitudinal in vivo imaging of demyelination after single OL laser ablation (in red), highlighting a myelin sheath (white arrowhead) that is stable up to D25 post ablation, but shows degradation profiles at D35 (insert) and no labelling at D37 (myelin labeling: AAV-*Mbp*:mem-tdTom). **b**: resident microglia surrounding the lost myelin sheath (*Cx₃cr1*$^{GFP/+}$ mouse), **c, top**: merged channels with high magnification of the inserts showing the transient alignment of microglial process with degenerating myelin at D35 (**c, bottom). d**: Additional examples of the local microglial response (arrowheads pointing at the lost myelin sheaths with microglial response). Displayed examples from 3 animals/areas. Display: 10µm maximum intensity projection. Scale bar: 20µm.

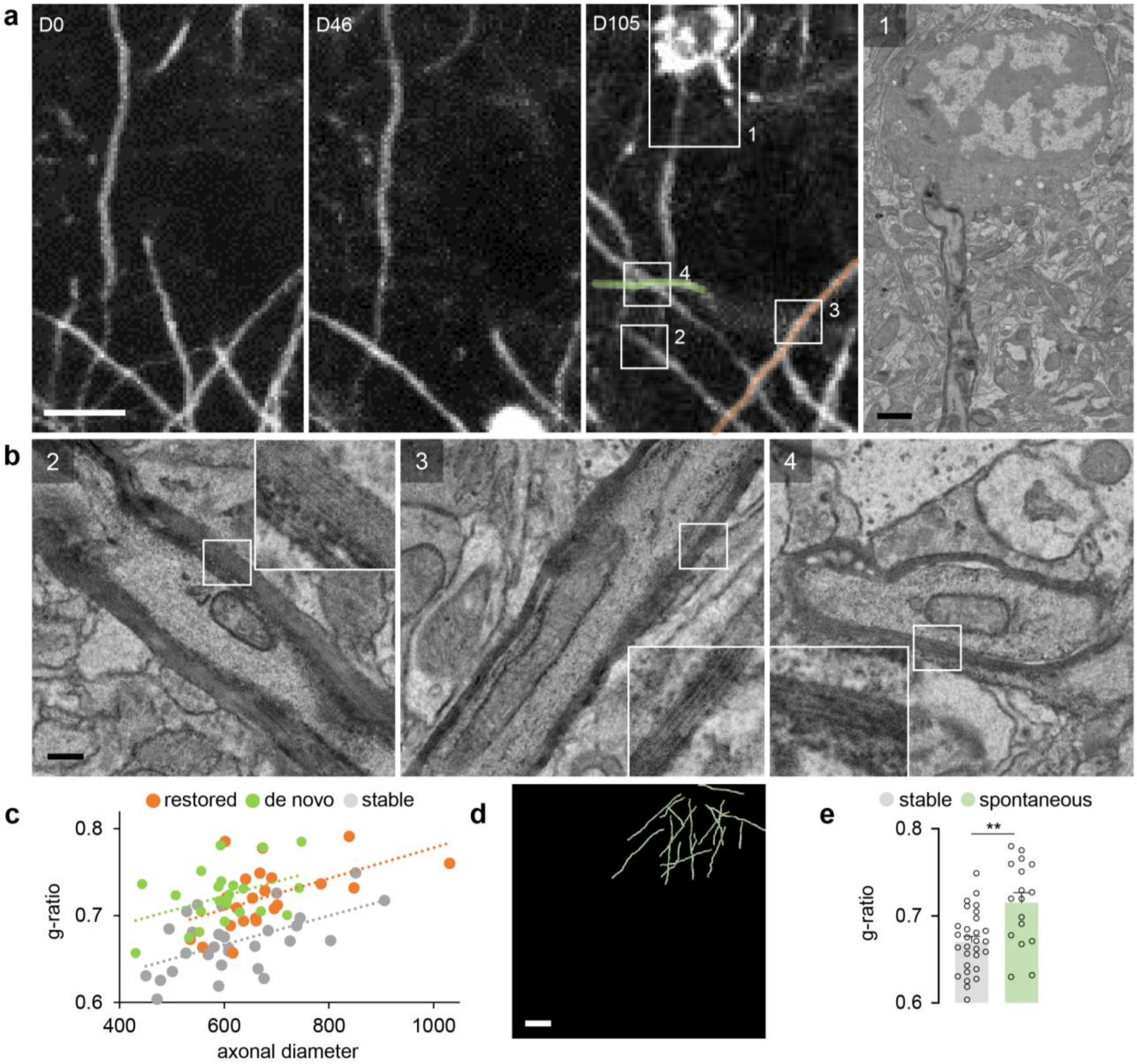

Supplementary Figure 5 | Ultrastructural integrity of newly formed myelin sheaths assessed by correlative high-resolution volume electron microscopy.

a: Longitudinal intravital imaging allowing the identification of restored (orange), de novo myelinated (green) and stable (white) segments after single OL ablation from a newly appearing OL (the morphology of the newly matured OL cell body is displayed from the marked area 1). **b**: Correlative EM at D105 of the three types of myelin sheath from the areas 2-4 in **a** (2: stable, 3: restored and 4: de novo), illustrating the regular membrane compaction and cytoplasmic space integrity of these sheaths. Inserts display the periodicity of the compacted myelin membrane. **c**: G-ratio measurements displayed in function of the axonal diameter for the restored (orange), de novo (green) and stable (grey) sheaths. **d**: 3D reconstruction of the spontaneously gained myelin internodes at 14 days after the OL ablation (before ablation induced myelin loss has been initiated). CLEM analysis was performed at D105, 90 days after first appearance of the spontaneously gained myelin. **e**: Average g-ratio stable internodes: 0.67 ± 0.006 and spontaneous gained internodes: 0.71 ± 0.015 (n=29 stable, n=17 spontaneous). Scale bars: 10µm for LM, 1 µm for EM in **a** and 200nm in **b** and 20 µm for 3D model in **d**. Statistics: mean ± SEM, in e: one-way ANOVA with Tukey's multiple comparison test including restored and de novo g-ratio datasets for testing, ** P<0.01.

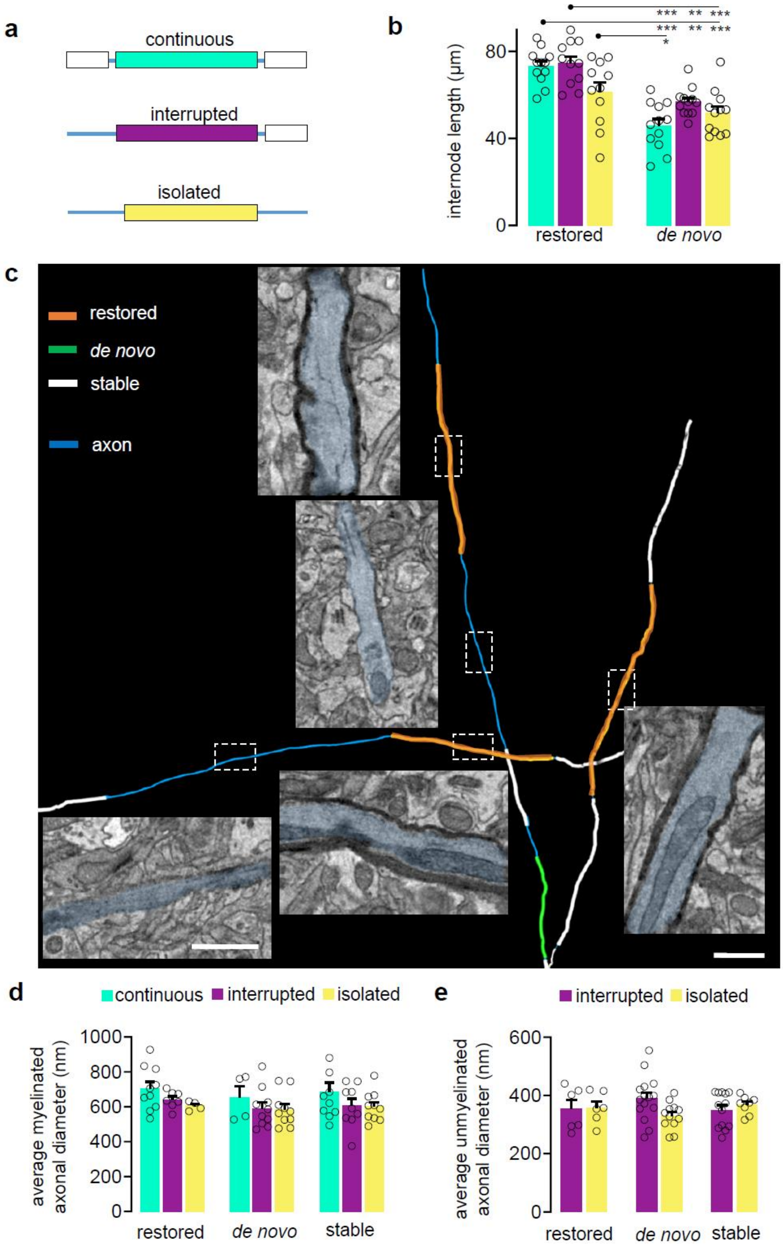

Supplementary Figure 6 | Myelin morphology related to internode type and myelination pattern.

a: Illustration of the three different internode types in the cortex with the continuous type having two neighboring sheaths, interrupted with an internode on only one side, and isolated without neighboring internodes. **b**: Average internode length of the restored and de novo fraction separated within the three internode types (n=12 areas from n=5 animals; mean ± SEM, one-way ANOVA with Tukey's multiple comparison test). **c**: 3D EM reconstruction of a restored segment from each internode type displaying similar axonal diameter, myelin thickness and unmyelinated axonal diameter (assessable only for interrupted and isolated segments). **d, e**: Quantification of the axonal diameter of restored, de novo and stable fraction depending on the internode type in myelinated (**d**: restored n=21, de novo n=24, stable n=29; mean ± SEM, one-way ANOVA) and unmyelinated section (**e**: consecutive to restored, n=12, to de novo n=27, to stable n=21; mean ± SEM, Kruskal-Wallis with Dunn's multiple comparisons test). *** P<0.001, **P < 0.01, *P<0.05. Scale bar, electron micrographs: 1µm, 3D rendering model, 20 µm.

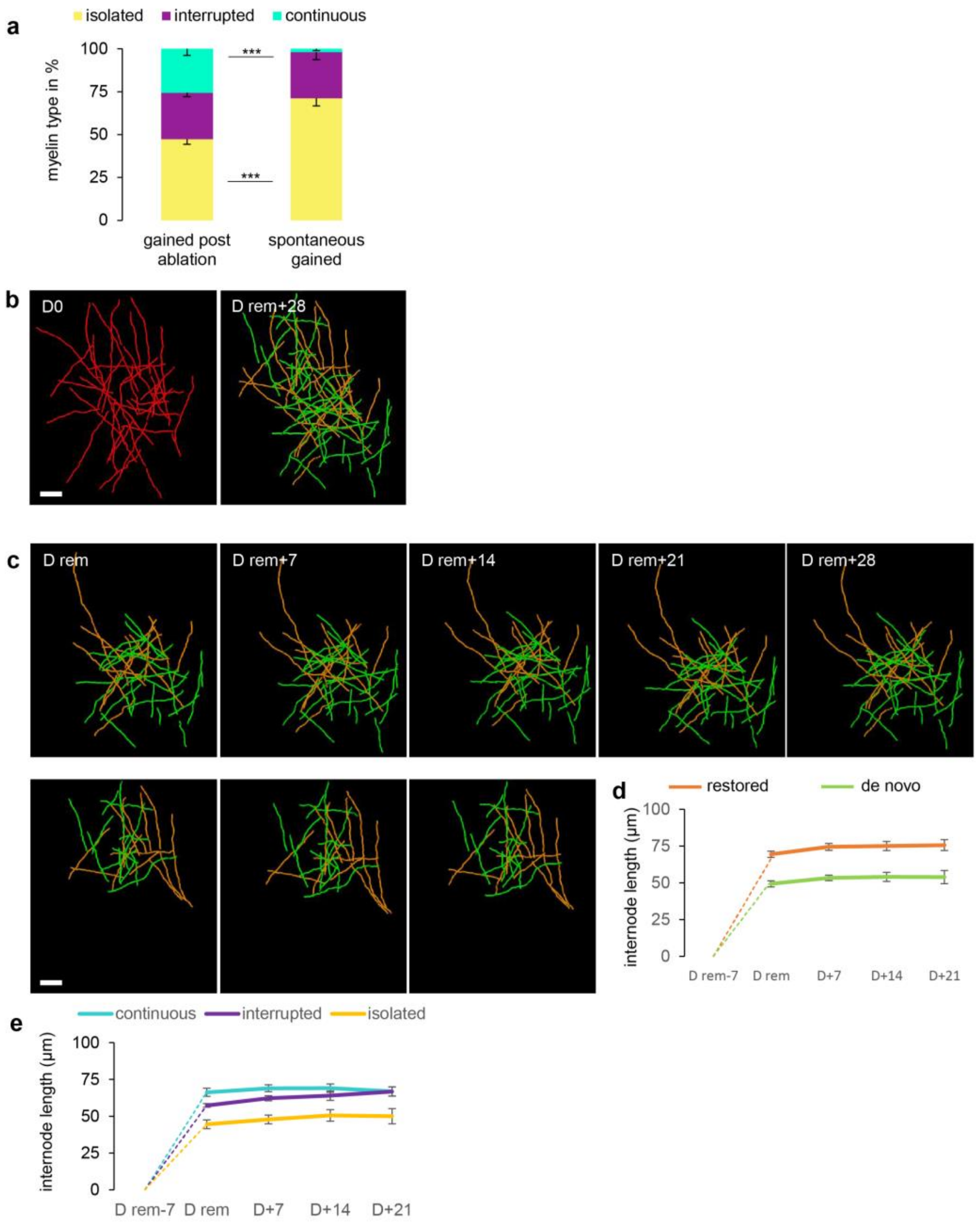

Supplementary Figure 7 | Stability of newly generated myelin sheath.

a: Integration pattern of the total gained internodes after ablation in comparison to the spontaneously gained internodes in control areas (continuous: 25.7 ± 3.8 and 1.9 ± 1.0, interrupted: 27.0 ± 2.2 and 26.8 ± 4.4, isolated: 47.3 ± 2.9 and 71.2 ± 4.5, n=12 and 10 areas from 5 and 6 mice, respectively for after ablation and in control, mean ± SEM, multiple t-test with Bonferonni-Dunn correction). **b**: Reconstruction of the lost myelin sheaths post ablation and the produced myelin sheaths from 2 newly appearing OL post ablation. **c**: Reconstruction of the gained myelin sheaths over time for each newly appearing OL. **d** and **e**: Quantification of myelin sheath length from appearance over time displayed for the restored and de novo fractions in **d**, and for the different myelin sheath types (continuous, interrupted and isolated) in **e** (n=7 OL from 3 animals/areas; mean ± SEM, in a: one-way ANOVA with Tukey's multiple comparison test). Statistics: *** P<0.001. Scale bar: 20µm.

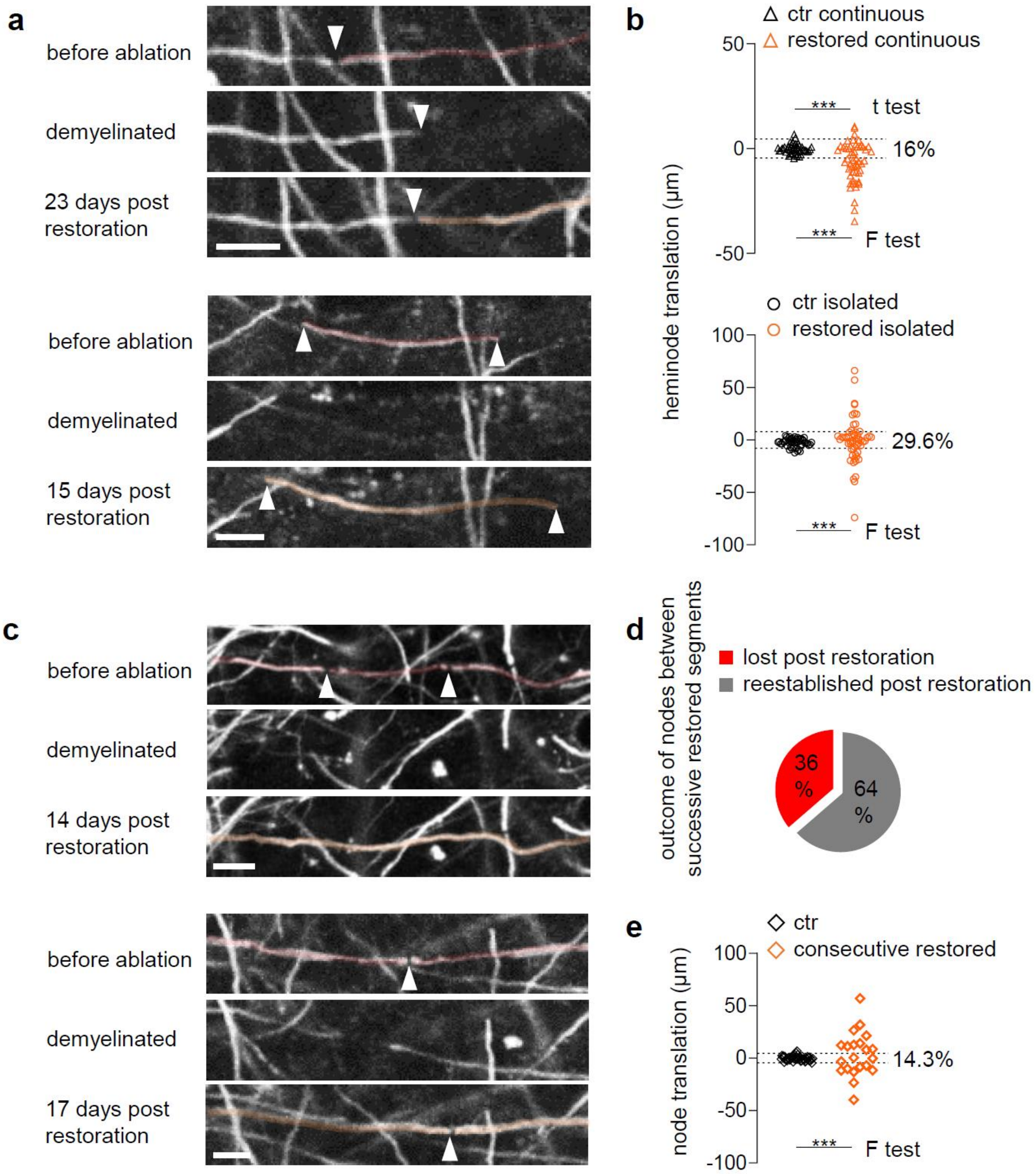

Supplementary Figure 8 | Node positon before and after demyelination-restoration cycle.

a: Node (white arrowhead) repositioning after demyelination and restoration for continuous (upper panel) and isolated (lower panel) and isolated myelin sheaths. **b**: Quantification of the heminode misplacement for a restored myelin sheath (continuous: n=46 heminodes in 5 areas from 5 animals for control and 52/7/5 for restored; isolated: n=50 heminodes in 5 areas from 5 animals for control and 54/9/5 for restored). Dotted lines plotted at 2 SD of control, i.e. stable nodes (ctr). **c**: Node lost (upper panel) and reestablished (lower panel) between successive restored myelin segments. **d**: Quantification of the lost and reestablished nodes between successive restored myelin segments. **e**: Quantification of the node misplacement for successive restored myelin sheath (continuous: n=46 segments in 5 areas from 5 animals for control and 21/10/6 for restored). Dotted lines plotted at 2xSD of ctr. Statistics: mean ± SEM, t-test and F-test, *** P<0.001. Scale bar: 10µm.
