# Supplementary material for: Myelin replacement triggered by single-cell demyelination in mouse cortex
Source: Nat Commun. 2020 Sep 29;11:4901. doi: 10.1038/s41467-020-18632-0 (PMC7525521; doi:10.1038/s41467-020-18632-0)
Supplement: Supplementary file 2 — Description of Additional Supplementary Files [file 41467_2020_18632_MOESM2_ESM.pdf]

Description of Additional Supplementary Files

Title: Supplementary Movie 1

Description: Intravital imaging of cortical OL in adult mouse. Representative area of the somatosensory cortex (layer 1), prior to single OL cell ablation in a Plp:GFP mouse, where internode morphology, OL processes and nodes of Ranvier are clearly identifiable. The imaged volume spans 60 µm in z and 203 µm in x/y (2.4 106 µm3) and exceeds the typical cylindrical volume of a cortical oligodendrocyte in our sample (~ 0.7 106 µm3).
