# Supplementary material for: Myelin replacement triggered by single-cell demyelination in mouse cortex
Source: Nat Commun. 2020 Sep 29;11:4901. doi: 10.1038/s41467-020-18632-0 (PMC7525521; doi:10.1038/s41467-020-18632-0)
Supplement: Supplementary file 4 — Reporting Summary [file 41467_2020_18632_MOESM4_ESM.pdf]

nature research

Corresponding author(s): Nicolas Snaidero

Last updated by author(s): Aug 20, 2020

Reporting Summary

Nature Research wishes to improve the reproducibility of the work that we publish. This form provides structure for consistency and transparency in reporting. For further information on Nature Research policies, see our Editorial Policies and the Editorial Policy Checklist.

Statistics

For all statistical analyses, confirm that the following items are present in the figure legend, table legend, main text, or Methods section.

n/a	Confirmed	
☐	☒	The exact sample size (*n*) for each experimental group/condition, given as a discrete number and unit of measurement
☐	☒	A statement on whether measurements were taken from distinct samples or whether the same sample was measured repeatedly
☐	☒	The statistical test(s) used AND whether they are one- or two-sided<br>*Only common tests should be described solely by name; describe more complex techniques in the Methods section.*
☒	☐	A description of all covariates tested
☐	☒	A description of any assumptions or corrections, such as tests of normality and adjustment for multiple comparisons
☐	☒	A full description of the statistical parameters including central tendency (e.g. means) or other basic estimates (e.g. regression coefficient) AND variation (e.g. standard deviation) or associated estimates of uncertainty (e.g. confidence intervals)
☐	☒	For null hypothesis testing, the test statistic (e.g. *F*, *t*, *r*) with confidence intervals, effect sizes, degrees of freedom and *P* value noted<br>*Give P values as exact values whenever suitable.*
☒	☐	For Bayesian analysis, information on the choice of priors and Markov chain Monte Carlo settings
☒	☐	For hierarchical and complex designs, identification of the appropriate level for tests and full reporting of outcomes
☒	☐	Estimates of effect sizes (e.g. Cohen's *d*, Pearson's *r*), indicating how they were calculated

Our web collection on statistics for biologists contains articles on many of the points above.

Software and code

Policy information about availability of computer code

Data collection	Two photon images were generated using an Olympus FV-RS microscope (Olympus, Japan)<br>EM micrographs were acquired on a Crossbeam Gemini 340 SEM (Zeiss, Germany)
Data analysis	Post-processing of the presented two-photon imaging and electron microscopy data were done using the open-source image analysis softwares, ImageJ/Fiji (version v1.53c) and IMOD (version 4.7). Statistical analysis were performed with Prism (version8.0)

For manuscripts utilizing custom algorithms or software that are central to the research but not yet described in published literature, software must be made available to editors and reviewers. We strongly encourage code deposition in a community repository (e.g. GitHub). See the Nature Research guidelines for submitting code & software for further information.

Data

Policy information about availability of data

All manuscripts must include a data availability statement. This statement should provide the following information, where applicable:

- Accession codes, unique identifiers, or web links for publicly available datasets
- A list of figures that have associated raw data
- A description of any restrictions on data availability

The Plp:GFP mouse line was generated by B.Z. to whom requests for the mouse line have to be directed. Cx3cr1GFP/+ and Thy1-GFPM mice are available from Jackson Laboratory (JAX 005582 and 007788, respectively). The plasmids to generate AAV-Mbp:mem-tdTom can be requested from the authors. Source data are provided in a separate Source Data File with this paper. The full datasets generated during and/or analysed during the current study are available from the corresponding author on reasonable request.

Field-specific reporting

Please select the one below that is the best fit for your research. If you are not sure, read the appropriate sections before making your selection.

- [x] Life sciences
- [ ] Behavioural & social sciences
- [ ] Ecological, evolutionary & environmental sciences

For a reference copy of the document with all sections, see nature.com/documents/nr-reporting-summary-flat.pdf

Life sciences study design

All studies must disclose on these points even when the disclosure is negative.

Sample size	No statistical methods were used to predetermine sample sizes, but sample sizes used in this study are comparable to those reported in previous publications using similar experimental design (see References 5, 18, 49, 61).
Data exclusions	Pre-established exclusion criteria were used in this study.<br>Areas showing deterioration of the imaging quality throughout the chronic imaging preventing new oligodendrocyte cell body identification (in all the data sets) or single internode resolution (in the data sets used in Figures 2-4) were excluded from the analysis.
Replication	We performed independently longitudinal intravital experiments across 3 or more independent experimental data sets. All attempts of replication allowed data collection. The correlative longitudinal intravital imaging to correlative volume electron microscopy (CLEM) was attempted three times. However, due to complex technical limitations of this experiment, only one CLEM experiment provided sufficient quality required to assess myelin sheaths ultrastructure within the entire correlated volume.
Randomization	Allocation of mice to experimental groups was random. However, due to the longitudinal nature of the imaging (data set correlated in time) and the binary type of experiment (obvious phenotype: ablated vs non ablated) no randomization was performed.
Blinding	Due to the longitudinal nature of the imaging (data set correlated in time) and the binary type of experiment (obvious phenotype: ablated vs non ablated) no blinding was performed.

Reporting for specific materials, systems and methods

We require information from authors about some types of materials, experimental systems and methods used in many studies. Here, indicate whether each material, system or method listed is relevant to your study. If you are not sure if a list item applies to your research, read the appropriate section before selecting a response.

Materials & experimental systems

n/a	Involved in the study	
x		Antibodies
	x	Eukaryotic cell lines
x		Palaeontology and archaeology
	x	Animals and other organisms
x		Human research participants
x		Clinical data
x		Dual use research of concern

Methods

n/a	Involved in the study	
x		ChIP-seq
x		Flow cytometry
x		MRI-based neuroimaging

Eukaryotic cell lines

Policy information about cell lines

Cell line source(s)	HEK293T obtained from ATCC https://www.lgcstandards-atcc.org/products/all/crl-3216.aspx?geo_country=de
Authentication	None of the cell lines used were authenticated.
Mycoplasma contamination	We confirm that all cell lines were tested negative for mycoplasma contamination.
Commonly misidentified lines<br>(See ICLAC register)	N/A

Animals and other organisms

Policy information about studies involving animals; ARRIVE guidelines recommended for reporting animal research

Laboratory animals	The following transgenic animals were used: Plp:GFP , Cx3cr1GFP/+ knock-in mice (ref 43), as well as Thy1:GFPM transgenic mice (ref 46) bred in our animal facilities on a C57BL/6 background. Both female and male animals from 3 to 6 months of age were included in

	experiments. The animals were housed at a temperature of 22+/-2°C with 50+/-15% of humidity levels and under a light/dark cycles of 12 hours.
Wild animals	No wild animals were used in the study.
Field-collected samples	No field-collected animals were used in the study.
Ethics oversight	All animal experiments were performed in accordance with the regulations of the relevant animal welfare acts (TierSchG) and protocols approved by the respective regional authorities (Regierung von Oberbayern).

Note that full information on the approval of the study protocol must also be provided in the manuscript.
